# A clean van der Waals interface between the high-*k* dielectric zirconium oxide and two-dimensional molybdenum disulfide

**DOI:** 10.1038/s41928-025-01468-1

**Published:** 2025-10-06

**Authors:** Han Yan, Yan Wang, Yang Li, Dibya Phuyal, Lixin Liu, Hailing Guo, Yuzheng Guo, Tien-Lin Lee, Minhyuk Kim, Hu Young Jeong, Manish Chhowalla

**Affiliations:** 1 Department of Materials Science and Metallurgy, University of Cambridge, Cambridge, UK; 2 School of Electrical Engineering and Automation, Wuhan University, Wuhan, China; 3 Diamond Light Source, Harwell Science and Innovation Campus, Didcot, UK; 4 Graduate School of Semiconductor Materials and Devices Engineering, UNIST, Ulsan, South Korea

**Keywords:** Electrical and electronic engineering, Electronic devices

## Abstract

Two-dimensional transition metal dichalcogenide semiconductors possess ideal attributes for meeting industry scaling targets for transistor channel technology. However, the development of scaled field-effect transistors (FETs) requires industry-compatible gate dielectrics with low equivalent oxide thicknesses. Here we show that zirconium oxide (ZrO_2_)—an industry-compatible high-dielectric-constant (*k*) oxide—can form a clean interface with two-dimensional molybdenum disulfide (MoS_2_). Photoelectron spectroscopy analysis shows that although silicon dioxide and hafnium oxide substrates introduce the doping of MoS_2_, ZrO_2_ exhibits no measurable interactions with MoS_2_. Back-gated monolayer MoS_2_ FETs using ZrO_2_ as a dielectric exhibit stable and positive threshold voltages of 0.36 V, subthreshold swings of 75 mV dec^−1^ and ON currents of more than 400 µA. We also use ZrO_2_ dielectrics to fabricate p-type tungsten diselenide FETs with ON-state currents of more than 200 µA µm^−1^. Atomic-resolution imaging of ZrO_2_ deposited on top of MoS_2_ reveals a defect-free interface, which leads to top-gated FETs with an equivalent oxide thickness of 0.86 nm and subthreshold swing values of 80 mV dec^−1^. The clean interface between ZrO_2_ and monolayer MoS_2_ allows the effective modulation of threshold voltage in top-gated FETs via gate metal work-function engineering.

## Abstract (web summary)

Zirconium oxide—an industry-compatible high-dielectric-constant oxide—can form a clean interface with two-dimensional molybdenum disulfide, allowing field-effect transistors to be created that offer small threshold voltages, low subthreshold swings and high ON-state currents.

## Main

Two-dimensional (2D) transition metal dichalcogenide (TMD) semiconductors could be used to build next generation sub-10-nm-channel field-effect transistors (FETs)^1–3^. The source–drain current and switching speed of FETs depends on the gate capacitance. Therefore, gate dielectrics with high dielectric constants (*k*) that can be scaled to very low equivalent oxide thicknesses (EOTs) are desirable. In bulk semiconductors, covalent bonding between the semiconductor and dielectric leads to low defect concentration at the interface, which minimizes threshold voltage instabilities^4^. Also, 2D TMDs form weak van der Waals bonds with dielectrics. Therefore, surface defect states on dielectrics remain unpassivated and can lead to charge transfer, interface states and electrostatic doping from dipoles. This results in variable 2D TMD FETs with substantial threshold voltage shifts, low ON-state currents and high subthreshold slopes. Therefore, pristine interfaces between 2D TMDs and dielectrics are essential for achieving high-performance FETs. The dielectric should also then offer a high *k* value to allow continued scaling and large band offsets (>1 eV) to prevent the injection of carriers from the semiconductor into its bands^4^.

Suitable dielectrics for 2D TMD FETs that are compatible with complementary metal–oxide–semiconductor technology have yet to be identified. Studies show that commonly used oxide dielectrics such as silicon dioxide (SiO_2_), aluminium oxide (Al_2_O_3_) and hafnium oxide (HfO_2_) heavily dope MoS_2_ (refs. ^5–7^), leading to n-type FETs operating in the depletion mode with large ON-state currents at zero gate voltage. Moreover, p-type FETs suffer from low ON-state hole currents because of electron doping from the dielectrics^8,9^. Interface hybridization between sulfur–oxygen at the MoS_2_/SiO_2_ interface can occur to passivate the oxide surface and the formation of new chemical bonds like Hf–S when MoS_2_ is in contact with the oxygen-deficient HfO_*x*_ surface^10,11^. These defects at the MoS_2_/dielectric interface lead to enlarged hysteresis and increased subthreshold swing (SS) values, along with large threshold voltage variation in the transfer characteristics.

Hexagonal boron nitride is often used as an inert dielectric substrate in 2D TMD devices, but it has a low dielectric constant^12^. Recent work has shown that it is possible to minimize the interactions between the dielectric and 2D TMDs by making free-standing strontium titanate (SrTiO_3_) membranes on which MoS_2_ is placed^13^. Yttrium oxide (Y_2_O_3_) thin-film transfer has also been reported^14^. A single-crystalline Al_2_O_3_ dielectric, formed via oxidation, has been reported for top-gated MoS_2_ FETs^15^. In these cases, the dry transfer of dielectrics leads to a van der Waals gap between the insulator and semiconductor to minimize interactions and form clean contacts^13–15^. Similarly, it has been shown that increasing the van der Waals gap between HfO_2_ and MoS_2_ can decouple interactions at the interface^11^.

Here we examine the interface between 2D MoS_2_ and the three most common industry-compatible dielectrics—SiO_2_, HfO_2_ and zirconium oxide (ZrO_2_)—using synchrotron photoelectron spectroscopy. The soft and hard X-ray photoelectron spectroscopy (XPS) analysis shows that SiO_2_ and HfO_2_ substrates lead to the doping of MoS_2_, but ZrO_2_ exhibits no measurable interactions with MoS_2_, which is consistent with density functional theory calculations. We create bottom-gated monolayer MoS_2_ FETs using ZrO_2_ as the dielectric and show that these devices offer stable and positive threshold voltages of 0.36 ± 0.3 V, SS values of 75 mV dec^−1^ and ON-state currents of more than 400 µA. We also create p-type 2D tungsten diselenide (WSe_2_) FETs with ON-state currents of more than 200 µA µm^−1^ using ZrO_2_ dielectrics. Atomic-resolution imaging of ZrO_2_ deposited on top of MoS_2_ shows a defect-free interface, and we create top-gated FETs with an EOT of 0.86 nm and SS of 80 mV dec^−1^. We also show that the clean interface between ZrO_2_ and monolayer MoS_2_ enables the effective modulation of threshold voltage in top-gated FETs by gate metal work-function engineering.

## Interface interactions between monolayer MoS_2_ and dielectrics

**Fig. 1:**
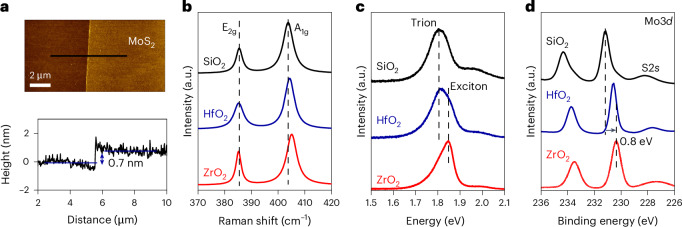
Monolayer MoS_2_ on SiO_2_, HfO_2_ and ZrO_2_ dielectric substrates. **a**, AFM image (top) and height profile (bottom) of the transferred MoS_2_. **b**, Raman spectra of MoS_2_ on SiO_2_, HfO_2_ and ZrO_2_. The redshift in the A_1g_ mode of MoS_2_ on HfO_2_ and SiO_2_ indicates electron doping from the substrate. **c**, PL spectra of MoS_2_ transferred on SiO_2_, HfO_2_ and ZrO_2_, showing the exciton-dominant peak on ZrO_2_ and trion-dominant peak on SiO_2_. HfO_2_ shows both trion and exciton peaks. **d**, Mo 3*d* peaks of MoS_2_ measured by XPS on different dielectrics, showing a Fermi-level shift of 0.8 eV on SiO_2_ and 0.3 eV on HfO_2_ with respect to ZrO_2_. MoS_2_ on SiO_2_ has a higher binding energy due to more electron doping from SiO_2_.

**Extended Data Fig. 1:**
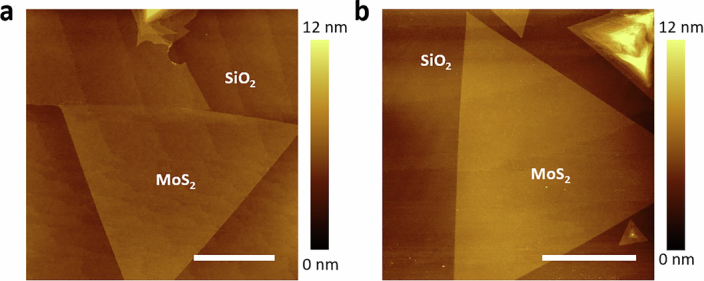
Clean transfer of CVD grown MoS_2_. **a** and **b**, Transferred CVD-grown MoS_2_ flakes on SiO_2_ using UV Ozone cleaned PDMS. Scale bar = 10 μm.

To ensure that the interfaces are not influenced by extrinsic artefacts, we use ultraviolet–ozone-cleaned polydimethylsiloxane as the medium for transfer of chemical vapour deposition (CVD)-grown MoS_2_. This leads to clean samples that are free of residue and are well adhered to the substrate (Methods). A typical atomic force microscopy (AFM) image of the samples used in this study and the corresponding height profile are shown in Fig. 1a, with a larger-scale AFM image shown in Extended Data Fig. 1. The measured thickness of 0.7 nm matches almost exactly with that of single-layer MoS_2_, suggesting that the samples are free of residue at the interface between the dielectric and MoS_2_.

**Extended Data Fig. 2:**
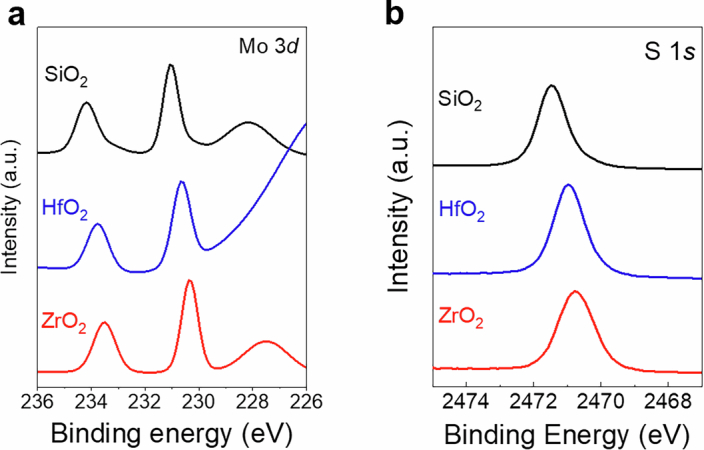
Hard X-ray photoelectron spectroscopies. **a**, XPS of monolayer MoS_2_ transferred on different substrate indicates more electron doping of MoS_2_ on SiO_2_ compared to HfO_2_ and ZrO_2_ collected. Blue curve shows an obvious Hf 4*d* peak at low binding energy due to higher probing depth. **b**, S 1*s* spectra collected with 3 keV beam energy also show higher binding energy for MoS_2_ on SiO_2_ compared to HfO_2_ and ZrO_2_.

The influence of dielectric substrates on 2D MoS_2_ was probed by Raman, photoluminescence (PL) and XPS measurements (Fig. 1b–d). The Raman spectra show that the E_2g_ peak of MoS_2_ remains relatively constant on different dielectrics, whereas the A_1g_ peak of MoS_2_ on SiO_2_ and HfO_2_ exhibits broadening and redshift compared with ZrO_2_, suggesting the electron doping of MoS_2_ from SiO_2_ and HfO_2_ (ref. ^16^). This is consistent with the PL spectra of monolayer MoS_2_ on SiO_2_ that show trion-dominant emission due to doping from the substrate and exciton-dominant emission on ZrO_2_ (Fig. 1c). The Mo 3*d* spectra of 2D MoS_2_ on different dielectrics were collected using soft X-rays (1 keV). The binding energy of Mo 3*d* from MoS_2_ on SiO_2_ is 0.5 eV higher than MoS_2_ on HfO_2_ and 0.8 eV higher than MoS_2_ on ZrO_2_, indicating that the Fermi level of MoS_2_ on SiO_2_ is 0.8 eV closer to the conduction band compared with MoS_2_ on ZrO_2_. Additionally, the Mo 3*d* and S 1*s* (intensity is higher than S 2*p*) spectra collected using 3-keV X-rays exhibit the same trend (Extended Data Fig. 2).

**Fig. 2:**
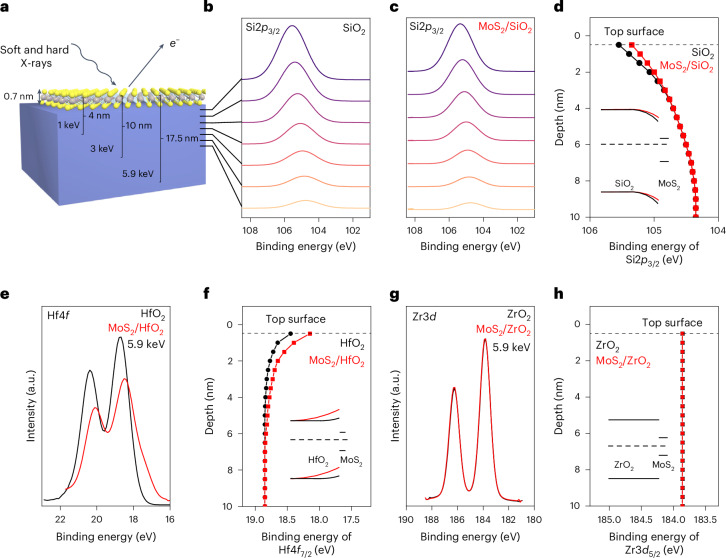
MoS_2_/dielectric interfaces measured by soft and hard XPS. **a**, Schematic of the photoelectron setup with probing depths labelled for SiO_2_. The probing depths for HfO_2_ are 2.5 nm at 1 keV, 5.5 nm at 3 keV and 10 nm at 5.9 keV. The probing depths for ZrO_2_ are 3.0 nm at 1 keV, 6.5 nm at 3 keV and 11.5 nm at 5.9 keV. **b**, Depth-resolved Si 2*p*_3/2_ spectra from the SiO_2_ substrate extracted from XPS modelling. **c**, Depth-resolved Si 2*p*_3/2_ spectra from MoS_2_ on the SiO_2_ sample extracted from XPS modelling. **d**, Summary of the binding energies of Si 2*p*_3/2_ at different depths for SiO_2_ and MoS_2_ on SiO_2_. Inset: schematic of the corresponding SiO_2_ surface band bending with and without MoS_2_ on top. **e**, Hf 4*f* spectra with and without MoS_2_ measured with 5.9-keV X-rays. **f**, Summary of the binding energies of Hf 4*f*_7/2_ at different depths derived from the XPS modelling for HfO_2_ and MoS_2_ on HfO_2_. Inset: schematic of the corresponding HfO_2_ surface band bending before and after contacting MoS_2_. **g**, Zr 3*d* spectra with and without MoS_2_ showing no measurable peak shift or broadening. **h**, Summary of binding energy positions of the Mo 3*d*_5/2_ at different depths for ZrO_2_ and MoS_2_ on ZrO_2_. The inset shows no band bending at the MoS_2_/ZrO_2_ interface.

To investigate the interface interactions between MoS_2_ and dielectrics, we collected the XPS spectra as a function of depth using a combination of soft and hard X-rays with energies of 1 keV, 3 keV and 5.9 keV (Fig. 2a). We extracted the valence band edge profile from the surface of the dielectric to a depth of ~17 nm for SiO_2_, 10 nm for HfO_2_ and 11.5 nm for ZrO_2_ (Methods). The binding energy shifts in XPS are caused by depth-dependent changes to the Fermi-level position within the bandgap, which can be determined to reconstruct the band edge energy profile at the MoS_2_/dielectric interface. By comparing the MoS_2_/dielectric interface with a pristine dielectric surface, the interactions between monolayer MoS_2_ and different dielectrics can be probed.

**Extended Data Fig. 3:**
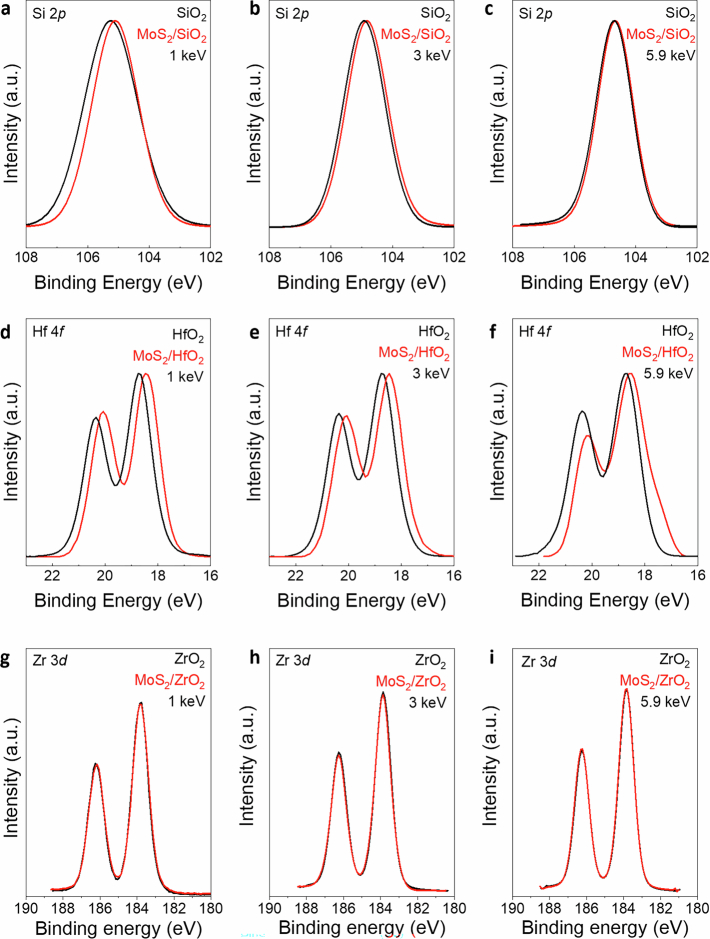
XPS of dielectrics and MoS_2_/dielectrics with different probing energy. **a–c**, Si 2*p* spectra of SiO_2_ and MoS_2_/SiO_2_ probed with 1 keV, 3 keV, and 5.9 keV, showing a higher binding energy in pristine SiO_2_ compared to SiO_2_ with MoS_2_ on top. This shift suggests that SiO_2_ induces n-type doping in MoS_2_. **d–f**, Hf 4*f* of HfO_2_ and MoS_2_/HfO_2_ probed with 1 keV, 3 keV, and 5.9 keV, similarly showing a higher binding energy in pristine HfO_2_ than in HfO_2_ with MoS_2_ on top, indicating n-type doping of MoS_2_ by HfO_2_. **g–i**, Zr 3*d* of ZrO_2_ and MoS_2_/ZrO_2_ probed with 1 keV, 3 keV, and 5.9 keV showing no measurable differences across all energies, suggesting minimal electronic interaction between MoS_2_ and ZrO_2_.

The depth-resolved Si 2*p*_3/2_ peaks from SiO_2_ with and without MoS_2_ on top are shown in Fig. 2b,c. The Si 2*p*_3/2_ peak of pristine SiO_2_ shifts to higher binding energies as the depth decreases. The Si 2*p*_3/2_ binding energies with MoS_2_ on top of SiO_2_ follow a similar trend. The binding energies of the Si 2*p*_3/2_ peak as a function of depth are summarized in Fig. 2d for two cases. Evidently, the band edge remains constant at depths of ≥6.5 nm. However, notable shifts in the Fermi level (binding energy) are observed at depths of <6.5 nm, causing the band to bend downwards. MoS_2_ on SiO_2_ does not influence the shifts in the Si 2*p*_3/2_ binding energy for depths beyond the top ~2 nm, where the binding energy shifts are lowered by the presence of MoS_2_, due to electron transfer from SiO_2_ to MoS_2_ at the interface. Figure 2d (inset) shows the energy band diagrams extracted from the XPS analysis with depth. It shows that a downward band bending of 1.2 eV ± 0.1 eV within the top ~6 nm in SiO_2_, which reduced to 1.0 eV ± 0.1 eV on the deposition of monolayer MoS_2_. The Hf 4*f* spectra from HfO_2_ and MoS_2_/HfO_2_ are summarized in Fig. 2e. The Hf 4*f* peaks recorded with 5.9-keV X-rays show substantial differences, indicating that the influence of MoS_2_ extends well into HfO_2_. Figure 2f and its inset show that the band bending for pristine HfO_2_ is upwards and it increases to ~0.4 eV ± 0.1 eV when MoS_2_ is deposited on top. The strong interface interactions are indicated by the substantial band bending, extending ~6 nm into HfO_2_. A direct comparison of Si 2*p*_3/2_ and Hf 4*f* binding is presented in Extended Data Fig. 3a–f. These results clearly show that SiO_2_ and HfO_2_ strongly perturb the MoS_2_/dielectric interface.

**Extended Data Fig. 4:**
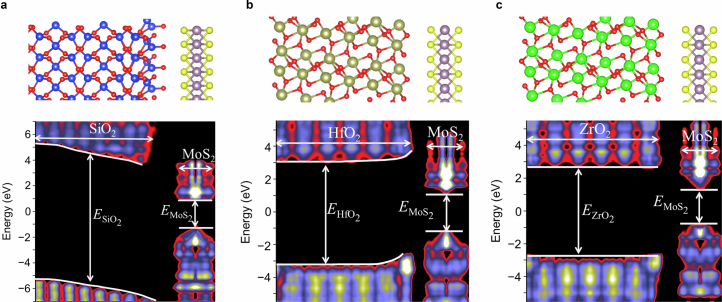
Calculated local density of states. **a**, Relaxed ball and stick model structures of SiO_2_ (silicon = blue and oxygen = red) and MoS_2_ (purple = Mo, yellow = sulphur) on top. LDOS for MoS_2_/SiO_2_ interface. E_SiO2_ is the band gap energy of SiO_2_. The valence and conduction bands bend downward due to interface dipole formed by unpassivated oxygen atoms. **b**, Relaxed ball and stick model structures of HfO_2_ (hafnium = gold and oxygen = red) and MoS_2_ (purple = Mo, yellow = sulphur) on top. The HfO_2_ surface reconstructs to form bridging oxygens. This leads to high electron density indicated by the bright white spot at valence band maximum near the surface. The states created by these bridging oxygens are at higher energy than the Hf-O bonds which causes the bands to bend upwards. **c**, Relaxed ball and stick model structures of ZrO_2_ (zirconium = green and oxygen = red) and MoS_2_ (purple = Mo, yellow = sulphur) on top. While bridging oxygens create high electron density at the valence maximum, their energies are comparable to the valence band energy so no band bending is observed. LDOS for MoS_2_/ZrO_2_ interface showing no band bending at the interface.

By contrast, the XPS analysis of ZrO_2_ shows no measurable surface states and deposition of MoS_2_ on top does not perturb the surface. The XPS spectra of the Zr 3*d* peak measured at 5.9 keV from pristine ZrO_2_ and with MoS_2_ on top are shown Fig. 2g. The Zr 3*d* spectra with other probing energies are summarized in Extended Data Fig. 3g–i. No measurable band bending is observed with or without MoS_2_ across all the probing energies. The Zr 3*d* binding energy as a function of depth is shown in Fig. 2h. These results indicate that the interface between MoS_2_ and ZrO_2_ is pristine without interactions. The local density of states (LDOS) of the MoS_2_/SiO_2_, MoS_2_/HfO_2_ and MoS_2_/ZrO_2_ interfaces were calculated using density functional theory (Methods and Extended Data Fig. 4). The results show that both SiO_2_ and HfO_2_ form interface dipoles with MoS_2_ due to unpassivated oxygen bonds on the SiO_2_ surface and reconstruction at the HfO_2_ surface involving oxygen. By contrast, the surface states in ZrO_2_ does not cause any band bending at the surface, consistent with our synchrotron photoelectron spectroscopy results.

## Device characteristics with different dielectrics

**Fig. 3:**
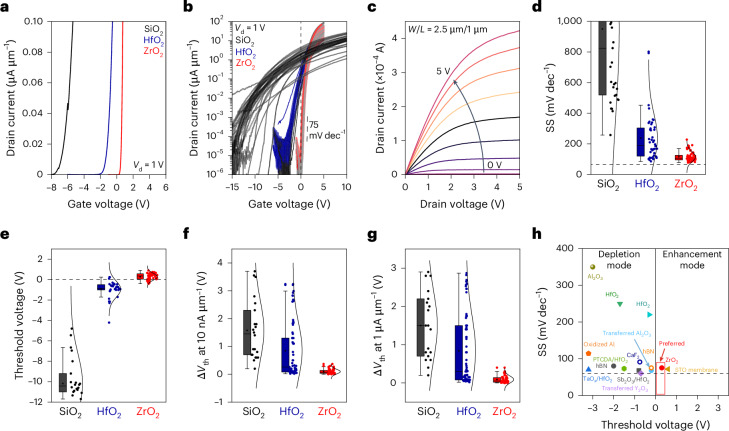
MoS_2_ FET device characteristics with SiO_2_, HfO_2_ and ZrO_2_ dielectrics. **a**, Transfer curves of MoS_2_ FETs plotted linearly to show the negative threshold voltages for MoS_2_ on SiO_2_ and HfO_2_ and positive threshold voltage for MoS_2_ on ZrO_2_. **b**, Logarithmic transfer characteristics of MoS_2_ FETs showing stable and low SS in devices with a ZrO_2_ dielectric compared with those with SiO_2_ and HfO_2_ dielectrics. **c**, Output curves of MoS_2_ FETs with ZrO_2_ showing an absence of barrier at the contacts and good saturation behaviour. **d**,**e**, Statistical distribution of SS (**d**) and *V*_th_ (**e**) of the MoS_2_ FETs. The ZrO_2_ dielectric shows low SS and small positive threshold voltage with a narrow distribution indicating reproducibility and consistency. **f**,**g**, Statistical distribution of threshold voltage variations (Δ*V*_th_) extracted at 10 nA µm^−1^ (**f**) and 1 µA µm^−1^ (**g**) for monolayer MoS_2_ FETs with SiO_2_, HfO_2_ and ZrO_2_ dielectrics. Statistics in **d**–**g** are based on 33 FETs with SiO_2_, 65 FETs with HfO_2_ and 72 FETs with ZrO_2_. Each box plot displays the 25th to 75th percentiles (box boundaries), median (central line) and mean (square symbol). Whiskers extend to a range of 1.5 times the interquartile range. The distribution curve scales the full data range. **h**, Summary of SS and *V*_th_ from monolayer MoS_2_ FETs, which shows that most MoS_2_ FETs devices reported in the literature work in the depletion mode^13,14,17–27^.

**Extended Data Fig. 5:**
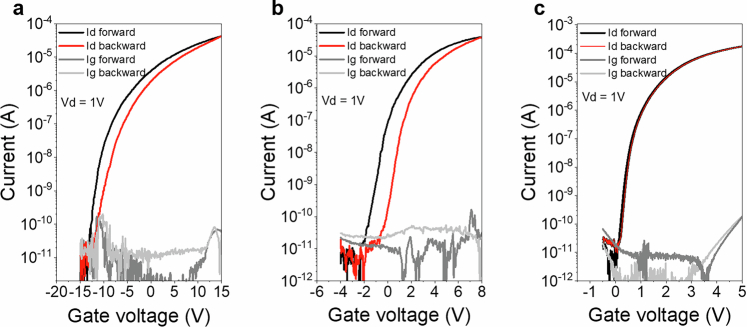
Hysteresis in monolayer MoS_2_ transfer curve. Forward (black) and backward (red) scans of MoS_2_ FETs with (**a**) SiO_2_, (**b**) HfO_2_, and (**c**) ZrO_2_ bottom gate dielectrics. It can be seen that the hysteresis of monolayer MoS_2_ on ZrO_2_ is much lower compared to SiO_2_ and HfO_2_ due to low interface defect states.

To investigate whether the pristine dielectric interface between ZrO_2_ and MoS_2_ can be translated into better and reproducible device performance, we performed electrical measurements on FETs fabricated using CVD-grown monolayer MoS_2_ as the channel with 90-nm SiO_2_, 40-nm HfO_2_ and 40-nm ZrO_2_ dielectrics as bottom-gate dielectrics (Fig. 3). The linear and logarithmic transfer characteristics of FETs using different dielectrics are shown in Fig. 3a,b, respectively. Figure 3a shows that FETs with SiO_2_ and HfO_2_ dielectrics operate in the depletion mode (channel is ON at zero gate voltage), whereas FETs with ZrO_2_ work in the enhancement mode (channel is OFF at zero gate voltage). The vast majority of monolayer MoS_2_ FETs reported in the literature work in the depletion mode in which a conductive channel is already formed at zero gate bias and require relatively high negative gate voltages to turn OFF the devices^17–27^. The transfer characteristics of one typical batch of over 50 monolayer MoS_2_ FETs fabricated with a ZrO_2_ dielectric (Fig. 3b) show that these devices completely turn OFF at zero gate voltage. By contrast, devices with HfO_2_ and SiO_2_ dielectrics exhibit high current at zero gate voltage, consistent with electron doping observed in the Raman, PL and XPS analyses. Our standard FET testing protocol involves taking forward and backward scans (Extended Data Fig. 5) and ensuring that the gate leakage current is ≪10^−9^ A. The output curves of monolayer MoS_2_ FETs with a ZrO_2_ dielectric are shown in Fig. 3c, showing ON currents of 423 µA with a channel length of 1 µm and width of 2.5 µm.

**Extended Data Fig. 6:**
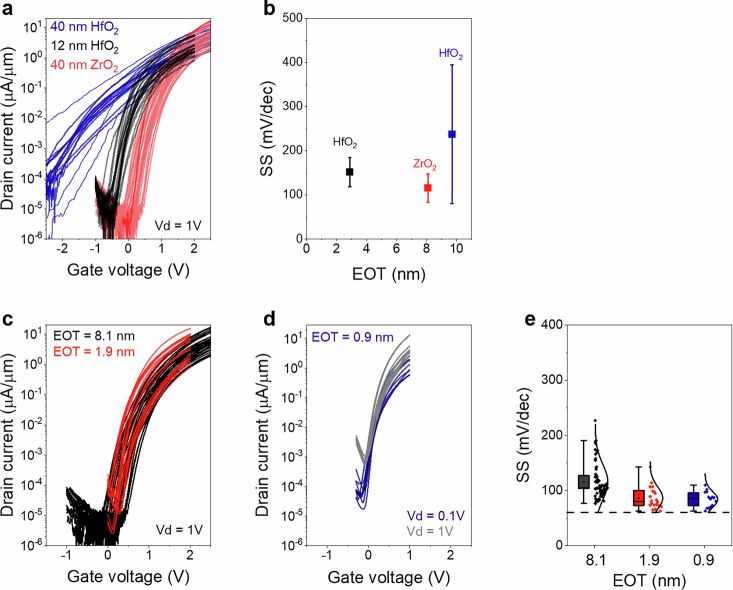
Thin dielectrics of HfO_2_ and ZrO_2_. **a**, Transfer curves of monolayer MoS_2_ FETs on 40 nm HfO_2_, 12 nm HfO_2_ and 40 nm ZrO_2_ dielectrics, respectively. MoS_2_ FETs with HfO_2_ dielectrics (both high and low EOTs) show negative threshold voltages. **b**, Comparison of SS values for HfO_2_ and ZrO_2_ dielectrics show that MoS_2_ FETs with ZrO_2_ dielectric exhibit low SS even with high EOT. Data points represent mean values, and error bars denote the standard deviation. Statistics in (**b**) are based on 18 FETs with 40 nm HfO_2_, 21 with 12 nm HfO_2_, and 28 with 40 nm ZrO_2_. **c**, Transfer curves of monolayer MoS_2_ FETs with ZrO_2_ dielectric for EOTs of 8.1 nm and 1.9 nm. **d**, Transfer curves of monolayer MoS_2_ FETs with ZrO_2_ dielectric for EOT of 0.9 nm at different source-drain biases. **e**, Statistical distribution of SS values for monolayer MoS_2_ FETs with ZrO_2_ dielectrics. Statistics in (**e**) are based on 72 FETs with 8.1 nm EOT, 20 with 1.9 nm EOT, and 14 with 0.9 nm EOT. Each box plot displays the 25th to 75th percentiles (box boundaries), the median (central line), and the mean (square symbol). Whiskers extend to a range of 1.5 times the interquartile range (IQR). The distribution curve scales the full data range.

**Extended Data Fig. 7:**
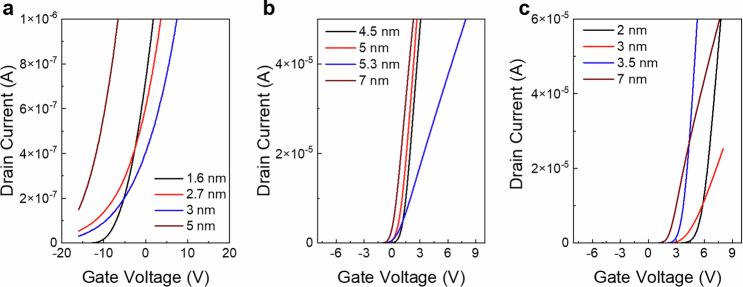
Device characteristics of multilayer MoS_2_ FETs on different dielectrics. **a**, Transfer curve of multilayer MoS_2_ FETs with SiO_2_ shows negative threshold voltage. **b**, Transfer curve of multilayer MoS_2_ FETs with HfO_2_ shows threshold voltage close to zero. **c**, Transfer curve of multilayer MoS_2_ FETs with ZrO_2_ shows positive threshold voltage.

The average SS values for bottom-gated MoS_2_ FETs with a ZrO_2_ dielectric were measured to be 115 ± 32 mV dec^−1^, considerably lower than those of devices on HfO_2_ (237 ± 157 mV dec^−1^) and SiO_2_ (950 ± 563 mV dec^−1^) dielectrics (Fig. 3d). ZrO_2_-based FETs demonstrated high reproducibility and consistent performance, achieving a minimum SS of ~75 mV dec^−1^. MoS_2_ FETs incorporating 12-nm HfO_2_ as the bottom dielectric (Extended Data Fig. 6a,b) demonstrated SS values of 151 ± 32 mV dec^−1^, which still fall short of the performance achieved by devices utilizing a 40-nm ZrO_2_ bottom dielectric. Moreover, bottom-gated MoS_2_ FETs with a ZrO_2_ dielectric scaled down to an EOT of ~1 nm demonstrated further improved SS values of 84 ± 14 mV dec^−1^ (Extended Data Fig. 6c–e). The highest mobility value of MoS_2_ FETs with ZrO_2_ was found to be 114 cm^2^ V^−1^ s^−1^, higher than MoS_2_ FETs with HfO_2_ (42 cm^2^ V^−1^ s^−1^) and SiO_2_ (26 cm^2^ V^−1^ s^−1^) dielectrics. Additionally, multilayer MoS_2_ FETs were fabricated with SiO_2_, HfO_2_ and ZrO_2_ dielectrics. The influence of dielectric doping from SiO_2_ and HfO_2_ on multilayer MoS_2_ is also observed in the transfer curves shown in Extended Data Fig. 7.

**Extended Data Fig. 8:**
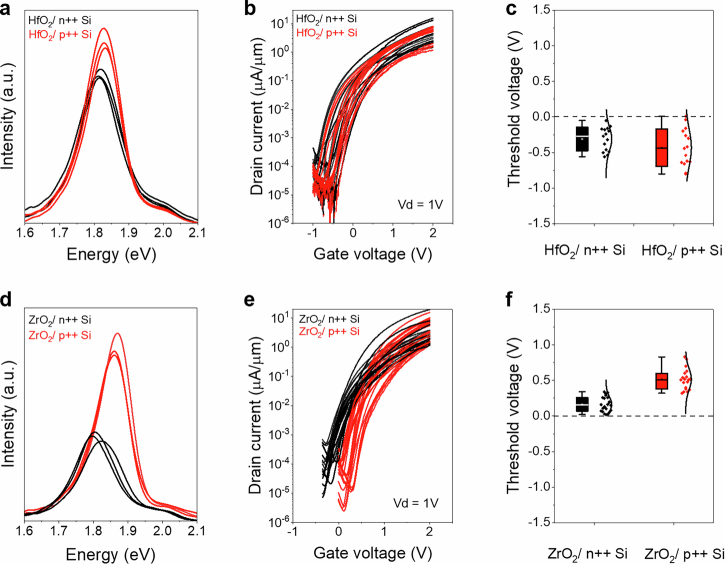
Influence of bottom gate work function on monolayer MoS_2_. **a**, PL spectra of CVD grown monolayer MoS_2_ transferred onto HfO_2_ grown on n++ Si and p++ Si. **b**, Transfer characteristics of monolayer MoS_2_ FETs with HfO_2_ dielectrics deposited n++ Si and p++ Si, showing minimal variation despite differences in gate electrode work function. **c**, Threshold voltages extracted at 10 nA/µm for MoS_2_ FETs with HfO_2_ dielectrics, comparing n++ Si and p++ Si gate electrodes. Statistics in (**c**) are based on 15 FETs for each gate electrode type. Each box plot displays the 25th to 75th percentiles (box boundaries), the median (central line), and the mean (square symbol). Whiskers extend to a range of 1.5 times the IQR. The distribution curve scales the full data range. **d**, PL spectra of CVD grown monolayer MoS_2_ transferred onto ZrO_2_ with different gate electrodes. Samples with n++ Si show trion dominant peak while p++ Si show more exciton dominant characteristics. **e**, Transfer characteristics of monolayer MoS_2_ FETs with different gate electrodes, incorporating ZrO_2_ dielectric layers. A lower threshold voltage is observed for metals with a lower work function. **f**, Threshold voltages extracted at 10 nA/µm for MoS_2_ FETs with ZrO_2_ dielectrics, comparing n++ Si and p++ Si gate electrodes. Statistics in (**f**) are based on 22 FETs with ZrO_2_ and n++ Si, and 19 with ZrO_2_ and p++ Si. Each box plot displays the 25th to 75th percentiles (box boundaries), the median (central line), and the mean (square symbol). Whiskers extend to a range of 1.5 times the IQR. The distribution curve scales the full data range.

We extracted the threshold voltage (*V*_th_) using the constant-current method (*I*_d_ = 10 nA µm^−1^) during gate voltage sweeps from negative to positive^28^. The *V*_th_ distribution of MoS_2_ FETs using SiO_2_, HfO_2_ and ZrO_2_ dielectrics is summarized in Fig. 3e. *V*_th_ for monolayer MoS_2_ FETs with ZrO_2_ was found to be 0.36 ± 0.3 V, whereas with HfO_2_, it was –0.93 ± 0.8 V. The *V*_th_ for MoS_2_ FETs with SiO_2_ is even more negative (–10.2 ± 3.9 V) due to electron doping. The distribution of *V*_th_ in Fig. 3e shows that all of our devices with ZrO_2_ show a small and positive voltage, indicating that MoS_2_ FETs with ZrO_2_ work in the enhancement mode—a desirable characteristic for digital circuits^29,30^. By contrast, the *V*_th_ for FETs with SiO_2_ and HfO_2_ is variable and negative. The influence of the bottom gate work function on *V*_th_ is shown in Extended Data Fig. 8. Hysteresis in FETs was quantified by extracting threshold voltage variations (Δ*V*_th_) at 10 nA µm^−1^ and 1 µA µm^−1^ (Fig. 3f,g). The results show that FETs with ZrO_2_ achieve lower hysteresis and improved stability. Although progress has been made on the realization of low SS FETs (Fig. 3h), here we show that it is possible to reproducibly make enhancement-mode FETs using industry-compatible high-*k* dielectrics.

**Extended Data Fig. 9:**
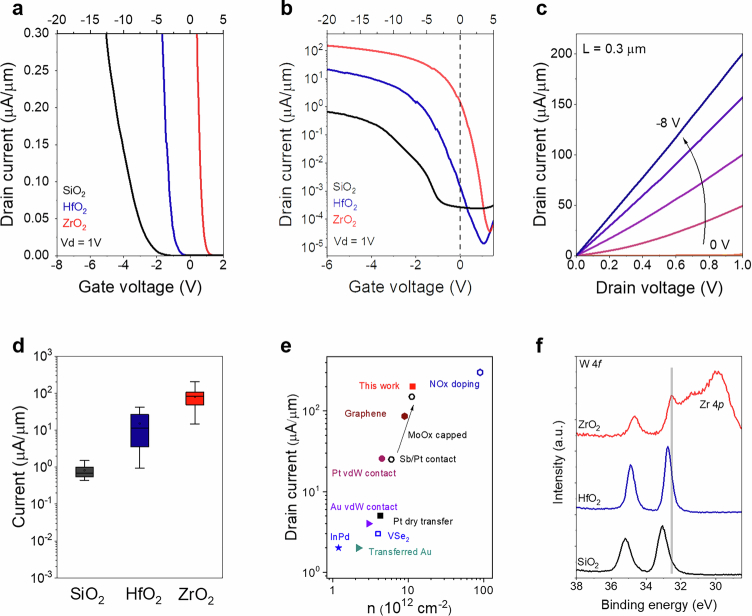
WSe_2_ P-type FETs with SiO_2_, HfO_2_ and ZrO_2_ dielectrics. **a**, Transfer curves of WSe_2_ FETs plotted linearly to show the negative threshold voltages for WSe_2_ FETs with SiO_2_ and HfO_2_ and positive threshold voltage for WSe_2_ FETs with ZrO_2_. The gate voltage scan range for SiO_2_ is shown on the top axis. **b**, Logarithmic transfer characteristics of WSe_2_ FETs with SiO_2_, HfO_2_ and ZrO_2_, highest hole current of 201 µA/µm for WSe_2_ on ZrO_2_. The gate voltage scan range for SiO_2_ is shown on the top axis. **c**, Output curves of WSe_2_ FETs with ZrO_2_ showing linear current-voltage characteristics. **d**, Hole current distribution of >20 WSe_2_ FETs on different dielectrics. ZrO_2_ dielectric shows the highest hole current compared to HfO_2_ and SiO_2_. Statistics in (**d**) are based on 7 FETs with SiO_2_, 18 with HfO_2_, and 17 with ZrO_2_. Each box plot displays the 25th to 75th percentiles (box boundaries), the median (central line), and the mean (square symbol). Whiskers extend to a range of 3 times the IQR. **e**, Drain current of P-type WSe_2_ FETs with values reported in the literature^8,34–41^^,42^. Solid symbols are for multilayer WSe_2_ FETs and empty symbols are for monolayer WSe_2_ FETs. **f**, W 4 *f* core level spectra of WSe_2_ on SiO_2_, HfO_2_, and ZrO_2_. The higher binding energy of W 4 *f* from WSe_2_ on SiO_2_ indicates higher electron doping of WSe_2_ from the dielectric.

To demonstrate the general applicability of ZrO_2_ dielectrics, we have also tested p-type FETs using multilayer WSe_2_ as the semiconductor channel on different dielectrics. It is well known that electron doping from SiO_2_ significantly reduces the hole current in WSe_2_ p-type FETs^8,9^ (Extended Data Fig. 9). The transfer characteristics of WSe_2_ FETs with van der Waals Pt contacts using different dielectrics show that the hole current in FETs with WSe_2_ on ZrO_2_ (~200 µA µm^−1^) is one order of magnitude higher than WSe_2_ FETs with HfO_2_ (~30 µA µm^−1^) and two orders of magnitude higher than WSe_2_ FETs with SiO_2_ (~1.5 µA µm^−1^) due to suppressed electron doping from ZrO_2_. The hole current realized on WSe_2_ FET with a ZrO_2_ dielectric is among the highest reported in the literature (Extended Data Fig. 9).

## Top-gated FETs and threshold voltage modulation

**Fig. 4:**
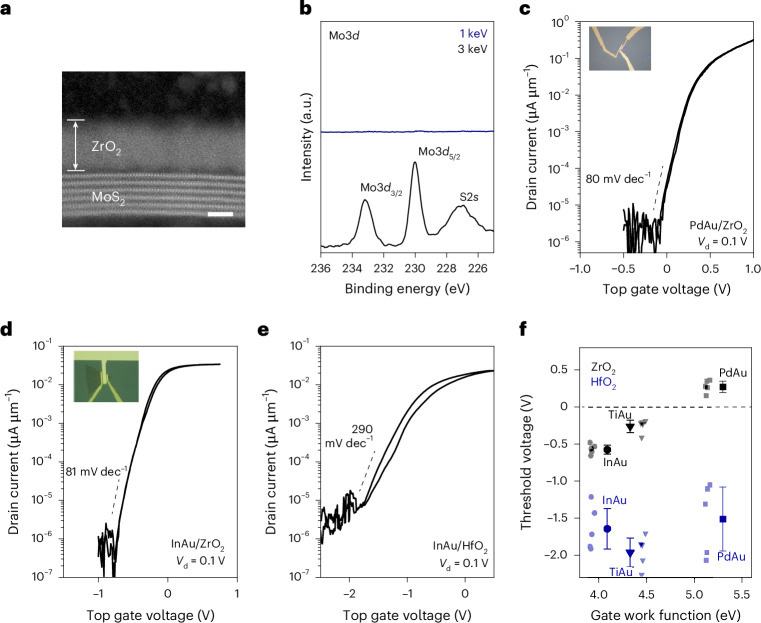
Top-gated device characteristics. **a**, Cross-sectional scanning transmission electron microscopy of ZrO_2_ deposited on multilayer MoS_2_, showing the defect-free interface between the dielectric and semiconductor. Scale bar, 2 nm. **b**, Mo 3*d* spectra measured with 1-keV and 3-keV X-rays, showing no detectable chemical interactions at the interface. The 1-keV spectra in blue do not show any Mo 3*d* signal as the X-rays only probe the top of ZrO_2_. The 3-keV spectra in black shows the Mo 3*d* spectra that is comparable to pristine MoS_2_, indicating that the deposition of ZrO_2_ has no chemical or structural impact on MoS_2_. **c**, Typical transfer characteristics of top-gated FETs based on multilayer MoS_2_ and ZrO_2_ as the top gate dielectric, and PdAu as the top gate metals. **d**, Typical transfer characteristics of FETs based on monolayer MoS_2_ and ZrO_2_ as the top gate dielectric, and InAu as the top gate metals. **e**, Typical transfer characteristics of FETs based on monolayer MoS_2_ and HfO_2_ as the top gate dielectric, and InAu as the top gate metals. **f**, *V*_th_ of FETs based on monolayer MoS_2_ with ZrO_2_ and HfO_2_ top gate dielectrics and gate metals with different work functions. Evidently, *V*_th_ can be modulated with a ZrO_2_ dielectric because of the defect-free interface with MoS_2_. Data points represent the mean values, and error bars denote the standard deviation. Statistics in **f** are based on five top-gated FETs for each combination of gate metal and dielectric.

To demonstrate the practical feasibility of a van der Waals dielectric, we fabricated top-gated FETs with a ZrO_2_ dielectric (EOT of 0.86 nm) on multi- and monolayer MoS_2_. The top dielectric/semiconductor interface was characterized by cross-sectional scanning transmission electron microscopy and XPS. Figure 4a shows that the interface between ~4.0 nm of ZrO_2_ on top of 2D MoS_2_ is pristine without any visible damage. Soft and hard XPS analyses of 4.0-nm ZrO_2_ grown on CVD-grown monolayer MoS_2_ are presented in Fig. 4b. The binding energy of Mo 3*d*_5/2_ in the ZrO_2_/MoS_2_/ZrO_2_ probed by hard X-rays is around 230.0 ± 0.1 eV, similar to that of the as-transferred MoS_2_ on a ZrO_2_ substrate, indicating that the deposition of the top dielectric does not induce chemical interactions at the interface.

**Extended Data Fig. 10:**
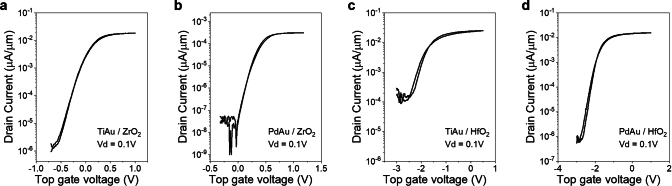
Typical transfer characteristics of top-gated FETs based on monolayer MoS_2_. **a**, Typical transfer characteristics of FETs based on monolayer MoS_2_, ZrO_2_ as the top gate dielectric and TiAu as top gate metals. **b**, Typical transfer characteristics of FETs based on monolayer MoS_2_, ZrO_2_ as the top gate dielectric and PdAu as top gate metals. **c**, Typical transfer characteristics FETs based on monolayer MoS_2_, HfO_2_ as the top gate dielectric and TiAu as top gate metals. **d**, Typical transfer characteristics of FETs based on monolayer MoS_2_, HfO_2_ as the top gate dielectric and PdAu as top gate metals.

Figure 4c shows the typical transfer characteristics of top-gated FETs based on multilayer MoS_2_ with a ZrO_2_ dielectric and Pd/Au gate metal. The results show negligible hysteresis, low subthreshold voltage and SS of ~80 mV dec^−1^. The transfer characteristics of top-gated FETs on CVD-grown monolayer MoS_2_ with different gate metals are shown in Fig. 4d (Extended Data Fig. 10). The most important result we obtain with top-gated devices is that the threshold voltage with the top-gated ZrO_2_ dielectric can be tuned from negative to positive. By contrast, FETs based on monolayer MoS_2_ with HfO_2_ as the top dielectric suffer from substantially higher SS values (292 ± 106 mV dec^−1^), large hysteresis and negative threshold voltages due to the defective dielectric/MoS_2_ interface (Fig. 4e and Extended Data Fig. 10). Clean ZrO_2_ on the MoS_2_ interface enables effective *V*_th_ modulation using gate metals with different work functions (Fig. 4f). Surprisingly, the *V*_th_ remains constant with the gate metal work function when HfO_2_ is used as the top gate dielectric and requires further careful theoretical analysis. These results highlight that the semiconductor/dielectric interface plays a vital role in enabling reliable threshold voltage modulation, minimizing hysteresis and achieving low SS values.

## Conclusions

We have shown that the doping of MoS_2_ is suppressed when ZrO_2_ is used as a high-*k* dielectric substrate. The clean MoS_2_/ZrO_2_ interface allows the realization of high-performance FETs that feature sub-1-nm EOT dielectrics and operate in the enhancement mode with positive and small threshold voltages, low SS values and high ON-state currents. Our results provide an insight into industrially compatible dielectrics for electronics based on atomically thin semiconductors.

## Methods

### Sample preparations

Monolayer MoS_2_ was obtained by CVD growth using MoO_3_ and sulphur as precursors. Here 5 mg of MoO_3_ powder was evenly distributed in an alumina boat and located at the centre of a single-zone tube furnace. SiO_2_ substrates, spin coated with 0.5 mg ml^−1^ of NaOH promoter, were placed face down on the MoO_3_ boat. Then, 60 mg of sulphur powder in another alumina boat was located 17 cm upstream at the edge of the furnace. Before starting growth, the tube was purged with 460 s.c.c.m. of N_2_ for 15 min at 150 °C. The N_2_ flow rate was then decreased to 60 s.c.c.m. The MoO_3_ source was heated to 720 °C and kept for 10 min, whereas the temperature of sulphur stabilized at around 230 °C. After the growth is done, the sulphur source was pulled out of the heating zone, and the tube was cooled down in 460 s.c.c.m. of N_2_ gas. Polydimethylsiloxane dry transfer technique is used to transfer the CVD-grown monolayer samples to the target dielectrics (Supplementary information).

Few-layered MoS_2_ and WSe_2_ samples were mechanically exfoliated from the bulk crystals (purchased from 2D Semiconductors) via the blue tape method. The target substrates are the same as mentioned above for the transferred CVD-grown samples.

Then, 90 nm of thermally grown SiO_2_, 40 nm of atomic-layer-deposition-grown HfO_2_ and 40 nm of atomic-layer-deposition-grown ZrO_2_ on boron degenerately doped silicon substrates were used in this study. The deposition conditions and characterizations of the dielectrics are given in the Supplementary Information.

### Soft and hard XPS measurements

Soft and hard XPS measurements were conducted at beamline I09 at the Diamond Light Source (UK) (beamtime under proposals SI30105-1, SI33391-1, SI32963-1 and SI38086-1). The spectra were collected through a Scienta Omicron EW4000 high-energy analyser. The beam size was around 15 μm × 35 μm. Also, 50-eV passing energy was used for soft X-rays (1,000 eV), whereas 200-eV passing energy was used for hard X-rays (3,000 eV and 5,900 eV). The binding energy scale was calibrated with the Au 4*f* core level of the gold electrodes on the samples as well as a gold foil on the sample holder. Radiation check was done on all the samples to confirm that there was no sample charging or beam damage during measurements. This included the repeated acquisition of core-level spectra five times. Furthermore, we proceeded to collect the actual spectra only if these radiation-checked spectra did not show any shift in binding energy or change in lineshape. The MoS_2_ samples used for the XPS measurements were CVD grown and transferred onto the corresponding substrates, same as those utilized in our device fabrication.

### Computational details

First-principles density functional theory calculations were carried out with the projector-augmented wave formalism implemented in the QuantumATK package^31^. The hybrid functional of Hyed–Scuseria–Ernzerhof (HSE06)^32^ was adopted with 85-Hartree cut-off energy for the plane-wave basis. Monoclinic HfO_2_ with an optimized lattice having *a* = 5.14 Å, *b* = 5.20 Å and *c* = 5.33 Å, ZrO_2_ with an optimized lattice having *a* = 5.18 Å, *b* = 5.25 Å and *c* = 5.37 Å and β-cristobalite SiO_2_ with an optimized lattice having *a* = *b* = 4.95 Å and *c* = 7.31 Å are chosen to match the hexagonal MoS_2_ monolayer with an optimized lattice having *a* = *b* = 3.17 Å. A 3 × 3 × 1 supercell model of MoS_2_ and 2 × 2 × 1 supercell model of the SiO_2_(001) surface were used to build the SiO_2_/MoS_2_ interface model. However, for the HfO_2_/MoS_2_ and ZrO_2_/MoS_2_ interface models, the $3\times \sqrt{3}\times 1$ supercell model of MoS_2_ was chosen to place on a 2 × 1 × 1 supercell model of the HfO_2_/ZrO_2_(001) surface. A vacuum-layer thickness of 20 Å was adopted in three interface models to avoid interactions between the imaging slab. The van der Waals correction with Grimme’s scheme was applied to describe the interface interactions^33^. The *k*-point sampling of 5 × 10 × 1 was used for the interface structural relaxations (converged to 0.01 eV Å^−1^) and 21 × 41 × 1 for the local density of states calculations.

### Measurements

Electrical measurements were carried out using a Keithley 4200 current–voltage system. PL and Raman data were collected using a 514-nm laser excitation focused through a ×100 objective lens. The spectra were taken at an incident laser power of 50 μW, which was sufficiently low to avoid any damage to the sample. AFM data were obtained using a Dimension Icon (Bruker) device in the peak-force tapping (ScanAsyst) mode.

## Supplementary information

Supplementary Information Supplementary Figs. 1–4, Table 1 and Discussion.

## Data Availability

The data that support the findings of this study are available from the corresponding authors upon reasonable request.
