# Supplementary material for: A clean van der Waals interface between the high-k dielectric zirconium oxide and two-dimensional molybdenum disulfide
Source: Nat Electron. 2025 Oct 6;8(10):906–12. doi: 10.1038/s41928-025-01468-1 (PMC12554778; doi:10.1038/s41928-025-01468-1)
Supplement: Supplementary file 1 — Supplementary Figs. 1–4, Table 1 and Discussion. [file 41928_2025_1468_MOESM1_ESM.pdf]

nature electronics

Supplementary information

https://doi.org/10.1038/s41928-025-01468-1

A clean van der Waals interface between the high-*k* dielectric zirconium oxide and two-dimensional molybdenum disulfide

In the format provided by the authors and unedited

A clean interface between high *k* dielectric and two-dimensional molybdenum disulfide

Table of contents:

S1. Clean sample preparation method

S2. Dielectric deposition and characterisation

S3. Synchrotron data modelling for interface depth profile

S4: Raman analysis of MoS_2 on different dielectric substrates

S1: Clean sample preparation method

Polydimethylsiloxane (PDMS) dry transfer technique (schematic shown in **Supplementary figure 1a**) is widely used to transfer chemical vapor deposition (CVD) grown samples to target dielectrics. However, the viscoelastic characteristic of PDMS prevents monolayer TMD from making intimate contact with the substrate and leaves residue. In this study, we optimised the transfer process reported by Jain *et al* [1] using UV Ozone cleaning the PDMS and use water induced capillary force to separate the MoS_2 from growth substrate (schematic shown in **Supplementary figure 1b**). The XPS results in **Supplementary figure 1c** shows that the carbon contamination is reduced and the PDMS becomes more rigid**.** The AFM images in **Supplementary figure 1d** clearly show that the height for PMDS transferred monolayer MoS_2 is ~2.5 nm. In contrast, the UV-cleaned PDMS transferred monolayer MoS_2 shows a height of ~ 0.7 nm. XPS was performed to confirm the absence of residue from MoS_2/ZrO_2 samples transferred by UV-cleaned PDMS (**Supplementary figure 1e**). The O 1*s* signal from PDMS transferred sample clearly show an additional peak at higher binding energy, indicative of silicone residue, whereas the UV-cleaned PDMS transfer process result in considerably less

residue. The influence of the transfer method on the doping characterisation is also shown in **Supplementary figure 1f**. The PL spectra collected from three distinct areas on one flake are inconsistent when PDMS transfer is used. In addition, the PL spectra is more exciton dominated for PDMS transferred monolayer MoS_2, whereas the UV-cleaned sample is more trion dominated due to electron doping from SiO_2 substrate.

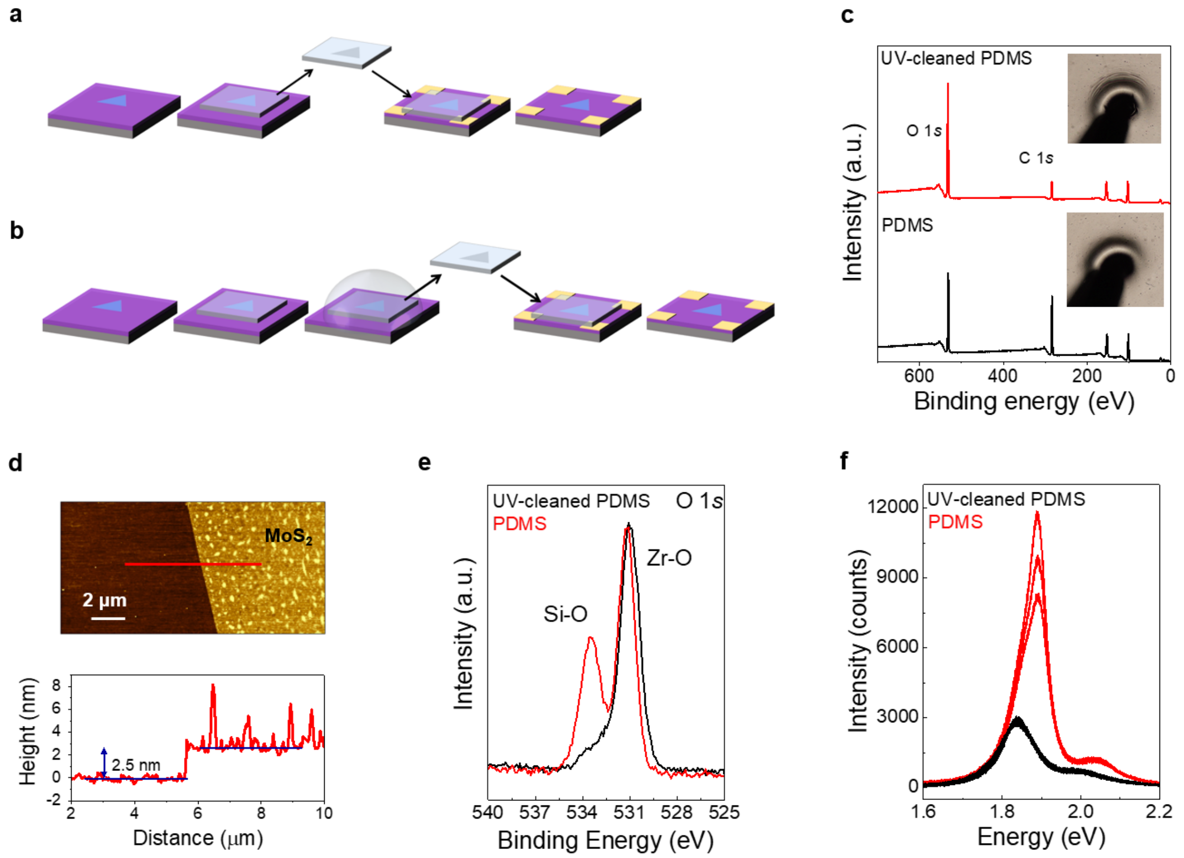

Supplementary figure 1. Clean transfer of CVD grown MoS_2. a, Schematic of PDMS assisted transfer method. **b**, Schematic of UV-cleaned PDMS transfer method. A water drop was introduced from the side of PDMS and driven by capillary force to transport underneath PDMS. The capillary force is strong enough to overcome the van der Waals force between MoS_2 and growth substrate. **c**, XPS survey spectra of PMDS and UV-cleaned PDMS clearly show that the C 1*s* peak is reduced after UV- Ozone cleaning. This gives less residue during

the transferring process. Inserts are photos of a tip brought in contact with PDMS and UV-cleaned PDMS, indicating the UV-cleaned PDMS is more rigid. **d**, AFM image of monolayer MoS_2 transferred onto new substrate using PDMS. The height profile shows that there is an air gap trapped between monolayer and substrate and the sample surface is not clean. **e**, O 1*s* of monolayer MoS_2 transferred onto ZrO_2 shows additional Si-O peak with PDMS transfer due to residue. **f**, PL of monolayer MoS_2 transferred on SiO_2 by PDMS and UV-cleaned PDMS. PDMS transferred samples are not consistent and show exciton dominant characteristics. UV-cleaned PDMS transferred samples give uniform PL signal and trion dominant signal due to intimate contact with SiO_2.

S2: Dielectric depositions and characterisations

90 nm of thermally grown SiO_2, 40 nm of ALD grown HfO_2, and 40 nm of ALD grown ZrO_2 on boron degenerately doped silicon substrates were used in this study. The ALD process was carried out using 60 sccm Ar as the carrier gas. Deposition of HfO_2 proceeded at 200 °C using TDMAHf and H_2O as precursors. The TDMAHf precursor container was heated to 75 °C. The pulse/purge times for TDMAHf and H_2O precursors were 60 ms/10 s and 250 ms/10 s, respectively. The ALD of ZrO_2 was carried out at 275 °C, and tetrakis(dimethylamino)zirconium (TDMAZr) and H_2O were the precursors. The TDMAZr precursor container was heated to 75 °C. The pulse/purge times for TDMAZr and H_2O were 60 ms/8 s and 250 ms/12 s, respectively.

The breakdown voltage of SiO_2 is over 20 V for 90 nm SiO_2 and 8 V for both HfO_2 and ZrO_2. The capacitance versus voltage (CV) measurements of SiO_2, HfO_2, and ZrO_2 were characterized with MIM structures, shown in **Supplementary figure 2**. The top contacts were made by metal deposition, with a shadow mask of 500-μm-diameter pattern. The CV

measurements were done at frequency of 10 kHz. The dielectric constant for HfO_2 and ZrO_2 are extracted to be 16 and 19, respectively.

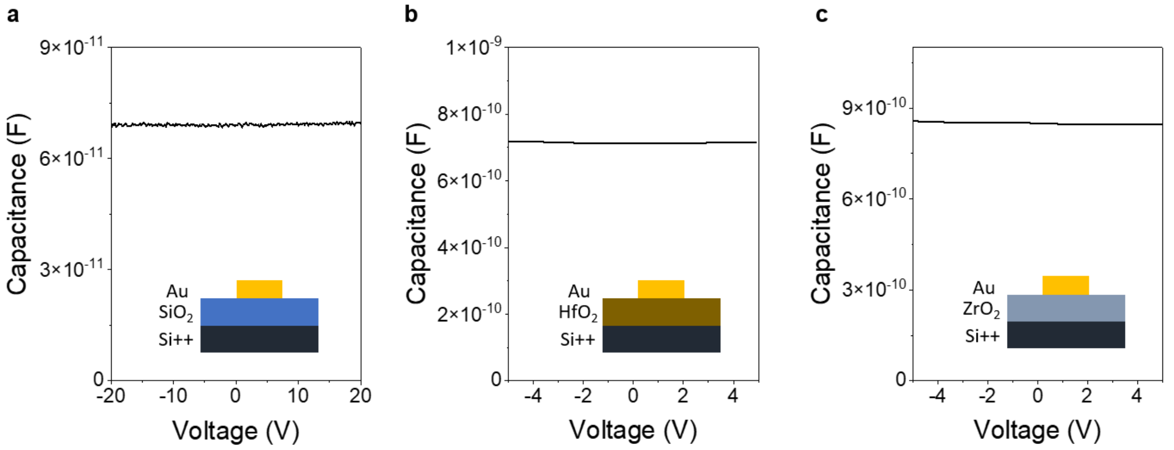

Supplementary figure 2. Capacitance- voltage measurements of the MIM structure for different substrates used in this study.

S3: Synchrotron data modelling

The method adopted in the present work for extracting band edge profiles is similar to those reported [2, 3]. The photoelectron inelastic mean free paths (λ) for different dielectrics and different X-ray excitation energies were obtained from NIST database [4]. For the same dielectric, a higher incident beam energy would result in a longer λ. The interface band bending profile can be extracted by soft and hard X-ray photoelectron spectroscopies.

For modelling the interface, the dielectrics were divided into a finite number of 0.5 nm-thick slices, of which the photoelectron signal (fitted with Voigt profiles) at depth *d* is damped by the factor of $e^{-\frac{d}{\lambda cos\theta}}$, where λ is the photoelectron inelastic mean free path and θ is the photoelectron emission angle with respect to the surface normal. Thus, the intensity proportion from each slice could be determined. The slice model captures around 90% of the total

photoelectrons, the last 10% is fitted with a single Voigt profile. In the modelling for each substrate, the line shape is shared across all slices and the binding energy position of each slice is shared across all photon energies. The binding energies of the core level in the individual slices are allowed to shift to reflect the Fermi level shifts and to reproduce the measured line shape. The Si 2*p* and Hf 4*f* spectra are deconvoluted first before modelling. The depth-resolved Si $2p_{3/2}$ peaks from SiO_2 with and without MoS_2 on top are shown in **Supplementary figure 3**. Hf 4*f* spectra from HfO_2 and MoS_2/HfO_2 are summarized in **Supplementary figure 4**. The Hf 4*f* peaks recorded with 5.9 keV X-rays show significant differences, indicating that the influence of MoS_2 extends well into HfO_2.

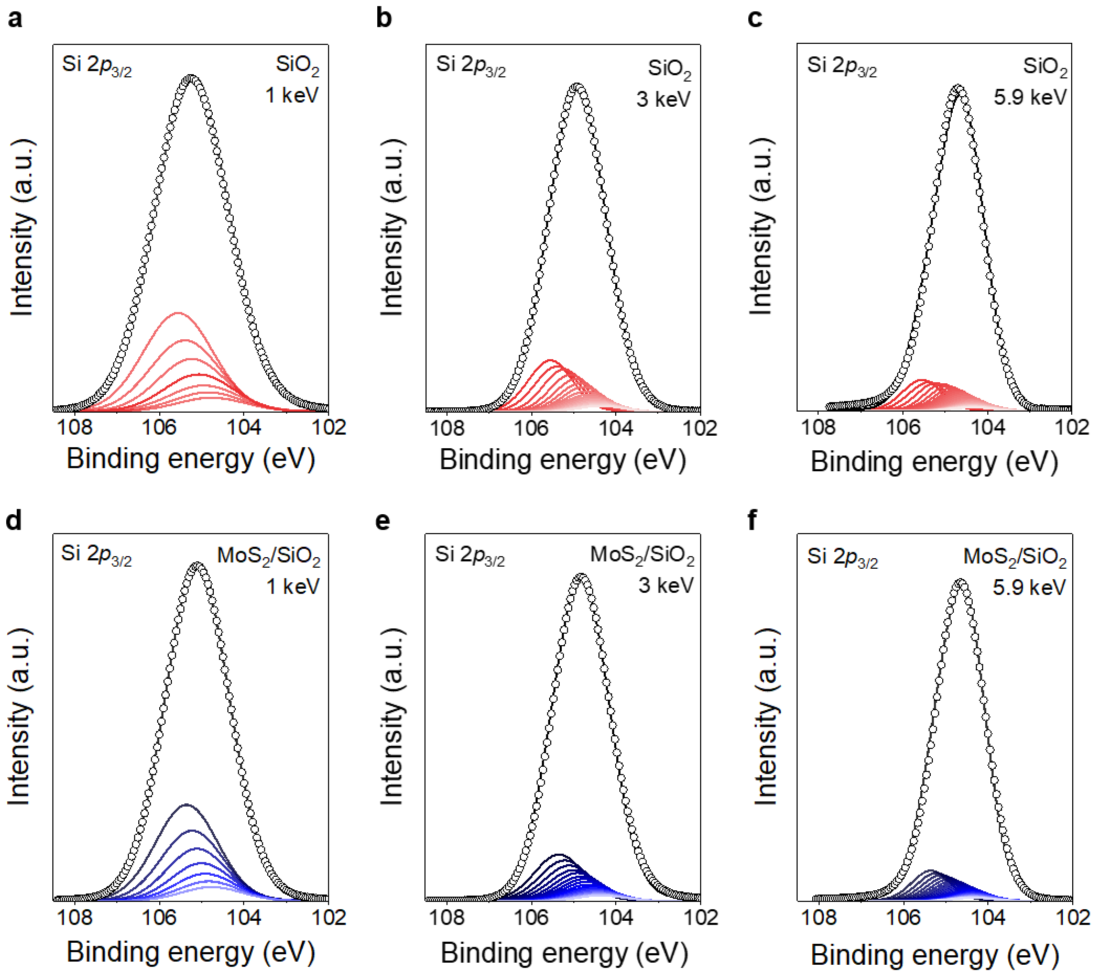

Supplementary figure 3. XPS of SiO_2 and MoS_2/SiO_2 with different probing energy. a - c, Si 2*p* of SiO_2 probed with 1 keV, 3 keV, and 5.9 keV. **d - f**, Si 2*p* of MoS_2/SiO_2 probed with 1 keV, 3 keV, and 5.9 keV. Qualitatively, the Si 2*p* spectra collected from top surface is broader and has higher binding energy compared to the spectra collected from bulk region. The Si 2*p* of SiO_2 is higher than Si 2*p* of MoS_2/SiO_2 at all probing energies due to more band bending at the surface.

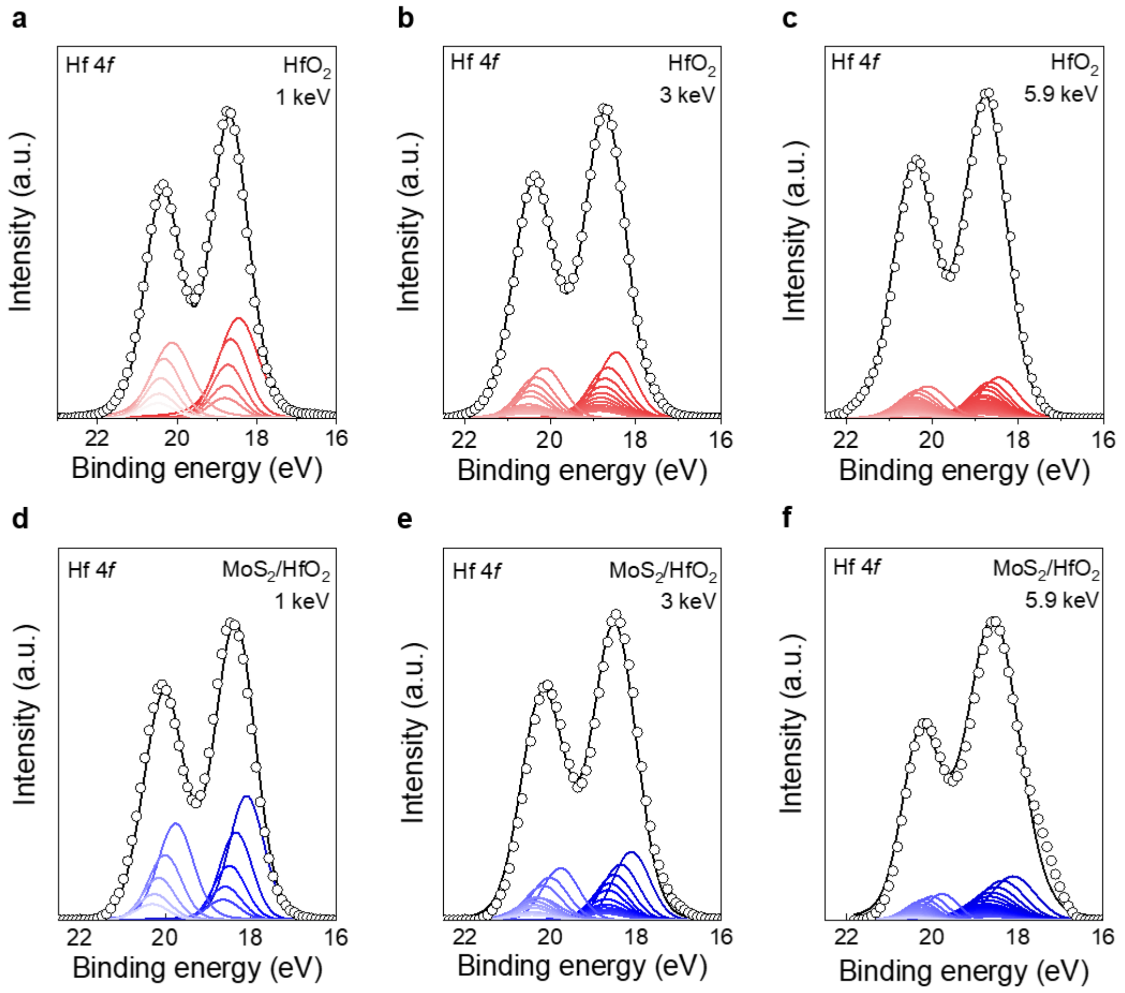

Supplementary figure 4. XPS of HfO_2 and MoS_2/HfO_2 with different probing energy. a - c, Hf 4*f* of SiO_2 probed with 1 keV, 3 keV, and 5.9 keV. **d - f**, Hf 4*f* of MoS_2/HfO_2 probed with 1 keV, 3 keV, and 5.9 keV. The peak shifts after contacting MoS_2 at lower probing energy but

gets broadening at higher probing energy, indicating the influence of MoS_2 extends to bulk HfO_2.

S4. Raman analysis of MoS_2 on different dielectric substrates.

Summary of Raman peak positions and full widths at half maximum (FWHM) for the A_{1g} mode of MoS_2 on different dielectric substrates. The values are extracted from five independent Raman measurements for each sample type, demonstrating the influence of the underlying dielectric on the doping of MoS_2.

	Peak position of A_{1g}	FWHM of A_{1g} peak
MoS_2 on SiO_2	403.82 ± 0.07 cm^{-1}	4.81 ± 0.61 cm^{-1}
MoS_2 on HfO_2	404.38 ± 0.32 cm^{-1}	4.76 ± 0.37 cm^{-1}
MoS_2 on ZrO_2	405.23 ± 0.45 cm^{-1}	4.19 ± 0.20 cm^{-1}

Reference:

1. Jain, A. et al. Minimizing residues and strain in 2D materials transferred from PDMS, ***Nanotechnology***, 29, 265203, (2018)
2. Sushko, P. V. & Chambers, S. A. Extracting band edge profiles at semiconductor heterostructures from hard-x-ray core-level photoelectron spectra. ***Sci. Reports*** 10, 1–10 (2020)
3. Stübinger, M. et al. Hard x-ray photoemission spectroscopy of $LaVO_3/SrTiO_3$: Band alignment and electronic reconstruction. ***Phys. Rev. B*** 103, 235128 (2021)
4. NIST Standard Reference Database 71 | NIST. doi:10.18434/T48C78.
